# *In silico* prediction and segregation analysis of putative virus defense genes based on SSR markers in sweet potato F1 progenies of cultivars ‘New Kawogo’ and ‘Resisto’

**DOI:** 10.5897/AJB2018/16724

**Published:** 2019-04-17

**Authors:** Alexander Ssamula, Anthony Okiror, Liat Avrahami-Moyal, Yehudit Tam, Amit Gal-On, Victor Gaba, Settumba B. Mukasa, Peter Wasswa

**Affiliations:** 1 Department of Agricultural Production, Makerere University, P. O. Box 7062, Kampala, Uganda; 2 Department of Plant Pathology and Weed Research, Agricultural Research Organization-The Volcani Center, Rishon LeZion 7505101, Israel

**Keywords:** *In silico*, segregation, simple sequence repeats (SSR) markers, sweet potato defense genes, virus

## Abstract

In sweet potato, an anti-virus defense mechanism termed reversion has been postulated to lead to virus freedom from once infected plants. The objectives of this study were to identify anti-virus defense genes and evaluate their segregation in progenies. Reference genes from different plant species were used to assemble transcript sequences of each sweet potato defense gene *in silico*. Sequences were used for evaluate phylogenetic relationships with similar genes from different plant species, mining respective defense genes and thereafter developing simple sequence repeats (SSRs) for segregation analysis. Eight potential defense genes were identified: RNA dependent RNA polymerases 1, 2, 5, and 6; Argonaute 1, and Dicer-like 1, 2, and 4. Identified genes were differentially related to those of other plants and were observed on different chromosomes. The defense genes contained mono-, di-, tri-, tetra, penta-, and hexa-nucleotide repeat motifs. The SSR markers within progenies were segregated in disomic, co-segregation, nullisomic, monosomic, and trisomic modes. These findings indicate the possibility of deriving and utilizing SSRs using published genomic information. Furthermore, and given that the SSR markers were derived from known genes on defined chromosomes, this work will contribute to future molecular breeding and development of resistance gene analogs in this economically important crop.

## INTRODUCTION

Sweet potato (*Ipomoea batatas* (L.) Lam.) production is severely affected by virus diseases that cause yield losses of up to 98% in individual plants (Gibson et al., 1998). Sweet potato is propagated vegetatively using vines as planting material, and farmers use vines from their own crop or traded with other farmers (Rachkara et al., 2017) to plant their gardens (Mukasa et al., 2003). The long-standing traditional practice of selecting healthy-looking vine plants as source material, coupled with low levels of symptomatic expression of many single virus infections, have led to the maintenance and proliferation of many viral pathogens (Rachkara et al., 2017). However, the expected high levels of viral prevalence throughout Uganda and consequent reduced yields have not materialized. It has been observed that previously infected field grown plants of East African sweet potato cultivars may become virus free (Adikini et al., 2016); this phenomenon is termed reversion. Similarly, a number of studies have reported that plants previously infected with *Sweet potato feathery mottle virus* became virus free (Green et al., 1988; Abad and Moyer, 1992; Gibb and Podovan, 1993; Gibson et al., 2014).

Gibson and Kreuze (2014) reviewed reversion in East African sweet potato cultivars, and suggested that it is a result of an RNA silencing mechanism triggered in plants by defense genes. The trigger for this plant defense response is the accumulation of viral dsRNA molecules in a replicative form or viral RNA secondary structures. Plant gene products, such as the RNA dependent RNA polymerases (RDRs), are part of the gene silencing machinery that independently synthesizes viral dsRNA in an amplification step for viral small RNA (21 to 24 nts) production. The dsRNAs are processed by Dicer-like (DCL) proteins to small RNAs, which are subsequently incorporated into the RNA-induced silencing complex with an Argonaute (AGO) protein that uses complementary small RNAs to target viral RNA (Baulcombe, 2004; Peragine et al., 2004; Hunter et al., 2016; Leibman et al., 2017). Thus, identification of putative defense genes involved in gene silencing is important in plant breeding for the management of virus diseases.

Defense genes have been studied and identified/predicted *in silico* in species such as *Nicotiana benthamiana* (Baulcombe, 2004), cucumber (Leibman et al., 2017), potato (Hunter et al., 2016), and cassava (Chellappan et al., 2004); however, the identity and nature of segregation and inheritance of defense genes in sweet potato require investigation. Different methods (webservers) of gene prediction and alignment have been developed. These include, GENOMESCAN (Yeh et al., 2001), AUGUSTUS (Stanke et al., 2004), Open Reading Frame Finder (Wheeler et al., 2003), GENIUS (Puelma et al., 2017), GENEMARK (Lomsadze et al., 2018), GENESCAN (Burge, 1998), Unipro UGENE (Okonechnikoy et al., 2012), CLC workbench (www.qiagenbioinformatics.com/products/clc-mainworkbench) among others. Further, plant based bioinformatics tools and databases have been developed, for instance SOL genomics Network (for Solanaceous plants) (Mueller et al., 2005) and Phytozome (for land plants and algae) (Goodstein et al., 2011). In a similar way, *Ipomoea* species tools have recently been developed. These include sweetpotato.plantbiology.msu.edu and sweet potato genome site (public-genomesngs.molgen.mpg.de/SweetPotato/). These are useful for understanding the genomics of polyploid sweetpotato. This study thus employed the SOL, Phytozome and *Ipomoea* species bioinformatics tools and GENESCAN, GENOMESCAN, Unipro UGENE and CLC gene prediction platforms, which are majorly open access prediction tools, which would be useful in countries of limited agricultural funding.

One of the methods of studying virus resistance inheritance is through genetic analysis (Mwanga et al., 2002). In this regard, simple sequence repeats (SSRs) are genetic markers that have received particular attention because they are highly informative, codominant, multi-allelic and are experimentally reproducible and transferable among related species (Mason, 2015). SSRs are used for various purposes. These include studies of diversity measured on the basis of genetic distance, evolutionary studies, constructing linkage maps, mapping loci involved in quantitative traits, estimating the degree of kinship between genotypes, marker-assisted selection, defining cultivar DNA fingerprints and estimating gene flow (segregation) in populations (Vieira et al., 2016).

Therefore, this study aimed to identify potential defense genes that may be responsible for reversion against virus infection, and evaluate their segregation patterns using SSR markers.

## MATERIALS AND METHODS

### Plant

Sweet potato cultivars „New Kawogo‟ and „Resisto‟ sourced from virus-free sweet potato collections at the Makerere University Agricultural Research Institute (MUARIK) and Namulonge Crops Resources Research Institute, respectively were used. The white fleshed „New Kawogo‟ is Ugandan in origin and is virus resistant (Gasura and Mukasa, 2010; Mwanga et al., 2016), while the orange fleshed „Resisto‟ from the USA is virus susceptible (Mwanga and Ssemakula, 2011). These cultivars were used as parents in a full diallel cross, with reciprocals considered (Griffing, 1956). Resulting seeds were harvested and planted in pots containing sterile potting mix that were then placed in an insect proof screenhouse at MUARIK. Imidacloprid and cypermethrin were applied weekly to control whitefly and aphid vectors of viruses. Each germinated seed was considered a progeny and was grown for 2 months prior to propagation using cuttings that were subsequently established in pots in an insect proof screenhouse at MUARIK.

### *In silico* prediction of defense genes

Defense gene transcript sequences from different plant species were obtained from the National Center for Biotechnology Information (NCBI) (www.ncbi.nlm.nih.gov) using Basic Local Alignment Search Tool (BLASTn) (Altschul et al., 1997), and used as references to derive similar gene sequences for sweet potato. The functions of the reference transcript sequences were verified using the Kyoto Encyclopedia of Genes and Genomes (www.genome.jp/kegg/), the Sol Genomics Network (SOL Genomics.net), and Phytozome (phytozome.net). The reference sequences were BLASTn searched in the Sweet potato genomics resource website (sweetpotato.plantbiology.msu.edu); this process identified homologous sequences within the genomes of the sweet potato relatives *Ipomoea trifida* and *Ipomoea triloba* (Wu et al., 2018). Then, these homologous sequences were used as a template to run a local BLASTn search within a database created in CLC genomic workbench software that was uploaded with a NOTEPAD file of transcript data and chromosomal locations sourced from the Sweet potato genome website (public-genomesngs.molgen.mpg.de/SweetPotato/).

Local BLASTn searches were conducted twice during *in silico* evaluation, where the first involved using high stringency parameters with the expectation value set at E^-10^; transcripts derived at this stringency level were denoted or assigned names depending on number of hits and level of homology. The second search was based on a low level of stringency, with the expectation value set at E^-6^, and names were assigned as before. This process revealed partial potential sweet potato virus defense gene transcripts and their respective chromosomal locations.

Further, the evolutionary relationship of each defense gene was estimated. This was done using the derived sweet potato virus defense gene transcripts and homologous gene transcripts (of different plant species) sourced from NCBI and sweetpotato.plantbiology.msu.edu. Phylogenetic trees were constructed using maximum likelihood method and following the Jukes and Cantor model (1969) in the CLC workbench. Observations were validated using Unipro UGENE software (Okonechnikov, 2012). Sequences used for rooting the phylogenetic tree were selected randomly.

Partial transcript sequences of sweet potato were also used as templates for mining full DNA sequences from the sweet potato genome website (public-genomesngs.molgen.mpg.de/SweetPotato/) using BLATn searching of the sweet potato genome (public-genomesngs.molgen.mpg.de/SweetPotato/; Yang et al., 2017). This process product of mining genomic DNA sequences on their respective chromosomes were screened for coding and non-coding regions using the MUSCLE (Edgar, 2004) sequence alignment program on the Unipro UGENE platform (Okonechnikoy et al., 2012). These regions were verified using online tools – GENESCAN, GENOMESCAN and CLC genomic workbench.

### Simple sequence repeats mining

DNA sequences within the coding regions were analyzed and screened for simple sequence repeats (SSRs) using WebSat software (wsmartins.net/websat) (Martins et al., 2009). This software was also used to generate SSR-based primers for analysis of segregation of the SSRs in the parental cultivars and their progenies. Outliers (sweet potato cultivars „Ejumula‟and „Tanzania‟ and the sweet potato relative *Ipomoea setosa*) were included. Previous work has shown that „Ejumula‟ is susceptible to virus infections (Mwanga et al., 2007), while „Tanzania‟ is moderately resistant (Gasura and Mukasa, 2010). The virus sensitive *I. setosa* is often used during virus diagnostics in sweet potato (Fuentes, 2010).

### Genomic DNA extraction

Genomic DNA of parents, progeny genotypes, and outliers was isolated using a modified version of the CTAB method (Maruthi et al., 2002). DNA quality was established using a NanoDrop-ND1000 spectrophotometer (Thermo Scientific, Bargal Analytical Instruments, Airport City, Israel), where DNA was diluted to 50 ng and used for downstream analysis. DNA was visualized on 1% agarose gel (VWN International) that was prepared by dissolving it in 0.5% Tris-Borate Acid (TBE) buffer, then warming in a microwave oven, followed by cooling over running tap water. Agarose gels were mixed with ethidium bromide (HyLabs, Rehovot, Israel), cast, and allowed to cool for 30 to 40 min. Then, 5 µl of diluted genomic DNA was mixed with 5 µl of loading dye (prepared using 0.25% bromophenol blue, 0.25% xylene cyanol, and 30% glycerol), loaded to the agarose gel, and run in the gel tank for 15 min at 120 V (Clever Scientific, Image Care, Kampala, Uganda).

### PCR amplification

Annealing temperature was optimized during amplification of the *in silico* derived primers using a gradient of eight temperatures on a PCR machine (Clever Scientific, Image Care, Kampala, Uganda). The optimal temperature that amplified polymorphic bands was used for subsequent evaluations. The 10 µl PCR master mix contained 3 µl of water, 5 µl of PCR mix (HyLabs Ready Mix [×2], HyLabs, Rehovot, Israel), 0.5 µl of each forward and reverse primer (10 pmol), and 1 µl of DNA (50 ng). The PCR conditions for SSR amplification were an initial denaturation at 94°C for 4 min, followed by 35 cycles of denaturation at 94°C for 30 s, annealing at 50°C for 45 s, extension at 72°C for 30 s, and final extension at 72°C for 10 min. A 3% agarose gel was used for visualization: the gel was warmed in a microwave oven, cooled, and then ethidium bromide was added and mixed. After cooling and setting, the gel was submerged in a gel tank containing 1x TBE buffer. 7 µl of PCR product was mixed with 2 µl of loading dye into the gel that was run at 50 V for 60 min.

### Band scoring

Bands were visualized on a gel documentation system (Clever Scientific, Image Care, Kampala, Uganda), where we first analyzed parental cultivars to evaluate and differentiate homozygous from heterozygous bands, according to Guo et al., (2015). A single band was considered to be homozygous at that locus or marker on the gene, while double bands were considered heterozygous. Therefore, primers that revealed double bands in one or both parents were used for progeny segregation analysis. Homozygous SSRs were also used to validate homozygosity in progenies. Bands were scored using binary counts of presence/absence (1/0) criteria.

### Segregation evaluation

Segregation was evaluated according to methods used by Zou et al. (2006) and Stift et al., (2008). SSRs that revealed double bands in parental genotypes were considered to be two markers of the same gene; thus, single bands had one marker of that gene. It was assumed that on crossing two markers (that is, A, a), the progeny followed the Mendelian segregation pattern, in a 1:2:1 ratio (1AA:2Aa:1aa). This was considered as disomic inheritance (Zou et al., 2006; Stift et al., 2008) and was revealed as three bands on the gel (Guo et al., 2015). Inheritance mode was classified following Zou et al., (2006), Stift et al., (2008), and Guo et al., (2015), where SSRs that revealed two bands were co-segregating, and zero, one, and four bands were considered as nullisomic, monosomic and tetrasomic inheritance, respectively. Chi-square goodness of fit analysis within XLSTAT (Addinsoft, 2017) was used to test the fit of segregation ratios of SSR markers to the disomic inheritance ratio of 1:2:1 at P ≤ 0.01.

## RESULTS

### Defense genes and SSRs

*In silico* prediction identified eight defense gene families of *I. batatas* (denoted *Ib*) (*IbRDR1*, *IbRDR2*, *IbRDR5*, *IbRDR6*, *IbAGO1*, *IbDCL1*, *IbDCL2*, and *IbDCL4*), located on 10 chromosomes (Table 1). There were six variants of *IbRDR1*; two of these (*IbRDR1a3* and *IbRDR1b1*) were not used (during segregation analysis), because they were highly (98%) homologous, and the other four variants were located on chromosomes 8 and 1 (Table 1). Two variants were found of *IbRDR5* located on chromosomes 14 and 11; two variants of *IbDCL1* located on chromosomes 1 and 9; and, three variants of *IbDCL2* located on chromosome number 12, 13, and 6. There were no variants of the remaining genes (Table 1).

**Table 1:** *In silico* prediction of sweet potato defense genes and chromosomal locations (according to genomic data published by Yang et al. (2017).

Gene	Chromosome	Variant identity
	8	*IbRDR1a1*
*IbRDR1*	8	*IbRDR1a2*
	1	*IbRDR1b2*
	1	*IbRDR1c*
*IbRDR2*	3	*IbRDR2*
>*IbRDR5*	14	*IbRDR5a*
	11	*IbRDR5b*
*IbRDR6*	10	*IbRDR6*
*IbAGO1*	3	*IbAGO1*
*IbDCL1*	1	*IbDCL1a*
	9	*IbDCL1b*
*IbDCL2*	12	*IbDCL2a*
	13	*IbDCL2b*
	6	*IbDCL2c*
*IbDCL4*	8	*IbDCL4*

### Abundance of SSRs

Mononucleotide, dinucleotide, trinucleotide, tetranucleotide, pentanucleotide, and hexanucleotide repeats were detected within the various coding regions of the defense genes. Pentanucleotide repeats were the most abundant (52.04%), while the least abundant were trinucleotide and tetranucleotide repeats (both were 4.09%) (Table 2). The highest proportion of repeats was observed in *IbRDR1a2* (44.67%), while the lowest was in *IbRDR6* (0.44%); and all forms of repeat were observed in *IbRDR1a2* (Table 2).

**Table 2:** Analysis of polymorphisms (repeats) in coding regions of the defense genes.

Gene	Variant	Number of repeats						Cumulative repeats	Proportion (%)
		Mono	Di	Tri	Tetra	Penta	Hexa		
*IbRDR1*	*IbRDR1a1*	0	1	0	0	2	3	6	2.46
	*IbRDR1a2*	20	11	8	8	37	25	109	44.67
	*IbRDR1b2*	0	0	0	0	3	1	4	1.64
	*IbRDR1c*	0	1	0	0	5	0	6	2.46
*IbRDR2*	*IbRDR2*	0	0	0	0	4	0	4	1.64
									
*IbRDR5*	*IbRDR5a*	0	1	0	0	1	0	2	0.82
	*IbRDR5b*	1	1	0	1	12	3	18	7.38
*IbRDR6*	*IbRDR6*	0	0	0	0	1	0	1	0.44
*IbAGO1*	*IbAGO1*	0	1	0	0	3	1	5	2.05
									
*IbDCL1*	*IbDCL1a*	2	3	1	0	11	3	20	8.19
	*IbDCL1b*	2	0	1	1	19	8	31	12.70
*IbDCL2*	*IbDCL2a*	1	0	0	0	10	2	13	5.33
	*IbDCL2b*	0	0	0	0	2	1	3	1.23
	*IbDCL2c*	0	0	0	0	1	1	2	10.82
*IbDCL4*	*IbDCL4*	1	1	0	0	16	2	20	8.19
Cumulative repeats	-	27	20	10	10	127	50	-	-
Abundance (%)	-	11.1	8.19	4.09	4.09	52.04	20.49	-	-

Mono: Mononucleotide repeats; Di: dinucleotide repeats; Tri: trinucleotide repeats; Tetra: tetranu-cleotide repeats; Penta: pentanucleotide repeats; Hexa: hexanucleotide repeats.

### Phylogenetic relationships of sweet potato defense genes to similar genes in other plant species

The phylogenetic relationship revealed that some putative sweet potato (*I. batatas* - *Ib*) defense genes or their variants had recently evolved and are either closely related to *I. trifida* or *I. triloba*; yet distantly related to those of other plant species.

The relationship of six species of *IbRDR1* varied. The *IbRDR1a1*, *IbRDR1a2, IbRDR1a3* evolved earlier than other *IbRDR1s*, though diverged from *I. triloba RDR1* variant 1. The *IbRDR1b2* and variant *IbRDR1a4* appeared to have recently evolved. All the *IbRDR1s* are related to variants of either *I. trifida*, *I. triloba* or *Ipomoea nil* RDR1 (Appendix Figure 1). The *RDR1* variants of other plant species like *Cucurbita* species, *Nicotiana* species, *Hevea brasillensis*, *Manihot esculenta* and *Oleo europaea* (Appendix Figure 1) are distantly related to *I. batatas RDR1* and its variants. Also, according to the phylogram, *I. batatas RDR2* recently evolved from *I. trifida* and *I. triloba*; though share a common ancestor

**Figure 1:**
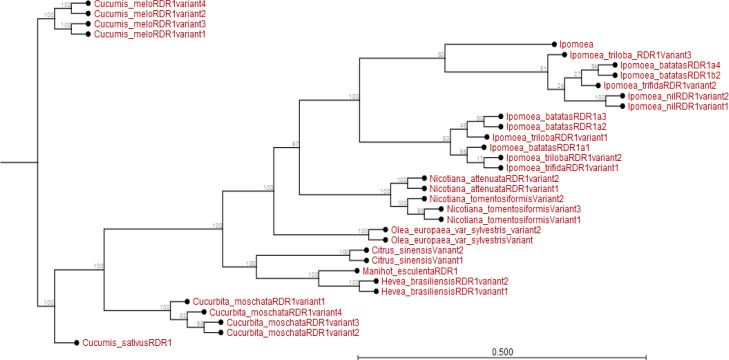
Phylogram showing evolutionary relationship of *Ipomoea batatas RDR1* and *RDR1* from plant species sampled from NCBI and sweetpotato.plantbiology.msu.edu.

(*I. nil RDR2*). All variants of *Ipomoea RDR2* diverged from the *RDR2* of *Nicotiana*, *Solanum* and *Capsicum* species. Also, *RDR2* of root crop *M. esculenta* and fruit crop *Vitis vinifera* evolved earlier than *I. batatas RDR2*. These *RDR2* also diverged extensively from *I. batatas RDR2* (Appendix Figure 2).

**Figure 2:**
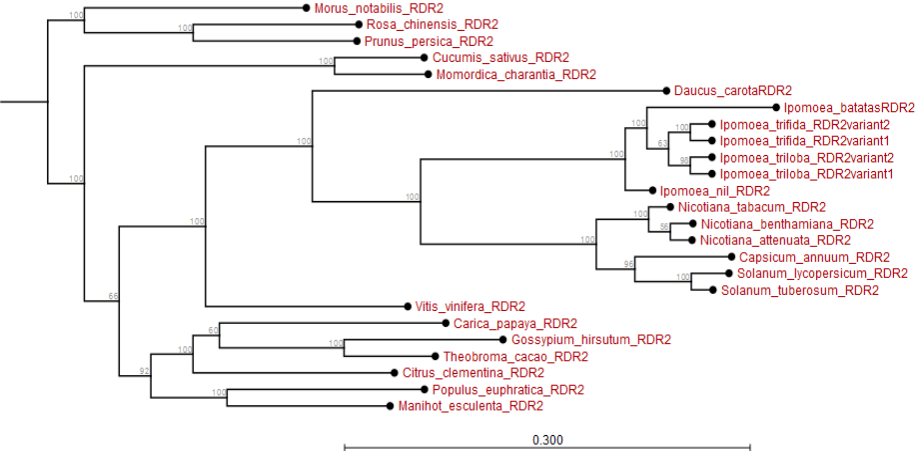
Phylogram showing evolutionary relationship of *Ipomoea batatas RDR2* and *RDR2* from plant species sampled from NCBI and sweetpotato.plantbiology.msu.edu.

When *RDR5* of different plants was estimated, it was observed that *RDR5* of all *Ipomoea* spp. evolved earlier than the *RDR5* of other plant species. The *IbRDR5b* evolved earlier than *IbRDR5a*. The *IbRDR5b* clustered with *I. triloba RDR5* variants yet *IbRDR5a* clustered with those of *I. nil* and *I. trifida* (Appendix Figure 3). On the other hand, the *RDR6* of *Nicotiana* and *Solanum* spp. evolved much earlier than that of *Ipomoea* spp. Regarding the respective *Ipomoea* spp., *I. batatas RDR6* evolved earlier than *RDR6* of *I. trifida*, *I. triloba* and *I. nil* (Appendix Figure 4).

**Figure 3:**
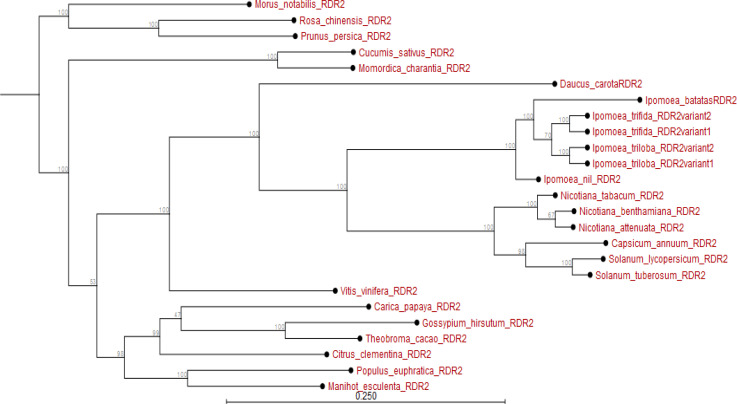
Phylogram showing evolutionary relationship of *Ipomoea batatas RDR5* and *RDR5* from plant species sampled from NCBI and sweetpotato.plantbiology.msu.edu.

**Figure 4:**
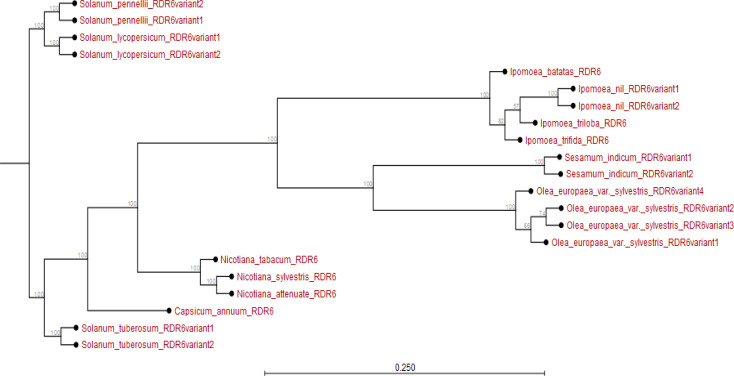
Phylogram showing evolutionary relationship of *Ipomoea batatas RDR6* and *RDR6* from plant species sampled from NCBI and sweetpotato.plantbiology.msu.edu.

The *I. batatas AGO1* and *I. trifida AGO1* are closely related and share *I. nil AGO1* as a phylogenetic ancestor. Further, whereas the *AGO1* of all *Ipomoea* spp. is related to the *AGO1* of *Nicotana* and *Solanum* spp., they diverged earlier from those of fruit trees like *V. vinifera* and *Citrus sinensis* among others (Appendix Figure 5). Additionally, with the exception of *DCL1* from *Solanum* spp. and *Nicotiana tabacum*, the *DCL1* of *Ipomoea* spp. has recently evolved. In particular, *I. batatas DCL1b* and *IbDCL1a* evolved earlier than *DCL1* of *I. trifida* or *I. triloba*; though highly related. The *DCL1* of other species sampled (for instance *Theobroma cacao*, *Hevea brasiliensis*, *M. esulenta* among others) evolved much earlier than *I. batatas DCL1* (Appendix Figure 6).

**Figure 5:**
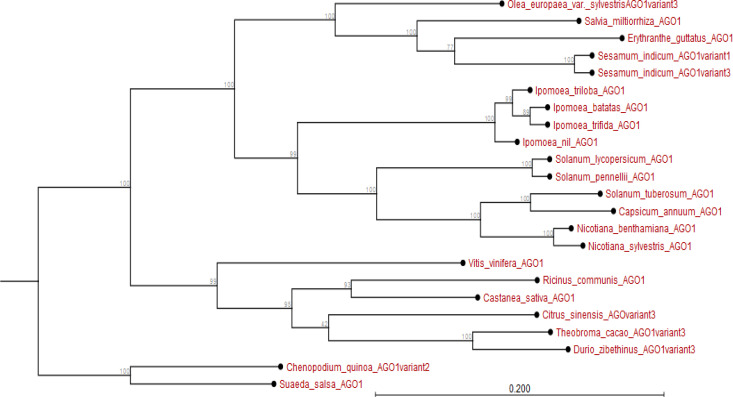
Phylogram showing evolutionary relationship of *Ipomoea batatas AGO1* and *AGO1* from plant species sampled from NCBI and sweetpotato.plantbiology.msu.edu.

**Figure 6:**
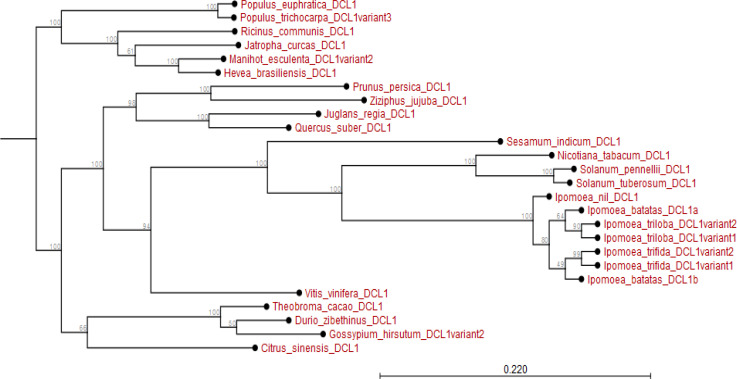
Phylogram showing evolutionary relationship of *Ipomoea batatas DCL1* and *DCL1* from plant species sampled from NCBI and sweetpotato.plantbiology.msu.edu.

The phylogram showed that *I. batatas DCL2b* and *IbDCL2c* diverged from *I. nil DCL2* and its variants. The *IbDCL2b* and *IbDCL2c* evolved earlier than *DCL2* from *I. triloba* and *I. trifida.* The *IbDCL2a* has recently evolved though related to *I. triloba* and *I. trifida*. The *DCL2* of plants like *Capsicum annum* and *Solanum lycopersicum* evolved earlier than that of *Ipomoea* spp. (Appendix Figure 7). The *I. batatas DCL4* is closely related to *I. triloba* and have recently evolved. The *DCL4* of other plant species evolved much earlier than *I. batatas DCL4.* The *DCL4* of *Solanum* and *Nicotiana* spp. evolved much earlier than those of *Ipomoea* spp. (Appendix Figure 8).

**Figure 7:**
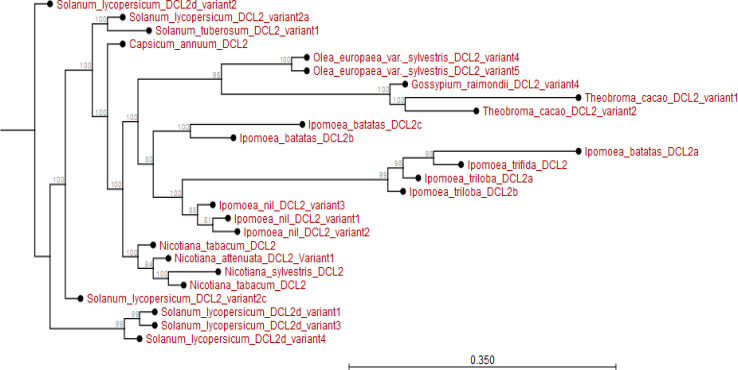
Phylogram showing evolutionary relationship of *Ipomoea batatas DCL2* and *DCL2* from plant species sampled from NCBI and sweetpotato.plantbiology.msu.edu.

**Figure 8:**
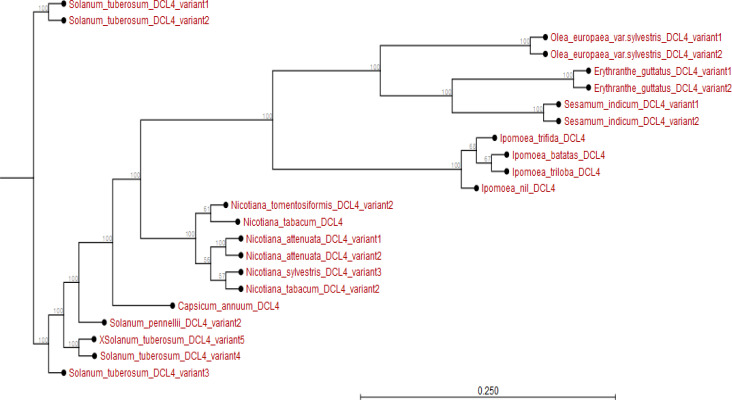
Phylogram showing evolutionary relationship of Ipomoea batatas *DCL2* and *DCL2* from plant species sampled from NCBI and sweetpotato.plantbiology.msu.edu.

### Segregation analysis of defense genes using SSRs

From a total of 222 SSR generated primers, 63 SSR primer sets were used in downstream analysis, from which nine showed heterozygous bands when evaluated in the parent cultivars, and were assumed to represent markers (Table 3).

**Table 3:** Heterozygous defense gene SSR variants and their respective markers.

Gene	Variant and chromosome (chr) location	Primer name	Repeat	Primer sequence (5′- 3′) Forward and Reverse	Identifier
	*IbRDR1a1* (Chr 8)	*IbRDR1a1_3*	(TTTATT)2	GGCCACATGGTAAATGAAGTAT GTGTTTTGAGGGCTGTTAATGT	Marker A
*ibRDR1*	*IbRDR1a2* (Chr 8)	*IbRDR1a2_17*	(TA)11	AAGCTGTAAGCACGGAGTAAAA AGAAGAAGAAGAAGAAGGAGGG	Marker B
	*IbRDR1a2* (Chr 8)	*IbRDR1a2_74*	(A)13	GCATTAGCGCATTACTGGTT AACACGATAAAGAAGATGAGGC	Marker C
	*IbDCL1a* (Chr 1)	*IbDCL1a_7*	(TTCAA)2	GGGTTGAAACACCTAGTAATGC AGCTGTGTGGAGGGTTAGTTTA	Marker D
	*IbDCL1a* (Chr 1)	*IbDCL1a_15*	(TA)10	GGGGTCATTTCTGTATGTGATT GTCCCTGCTTCAAAGGTAAGAT	Marker E
*IbDCL1*					
	*IbDCL1b* (Chr 9)	*IbDCL1b_23*	(AGTAGC)2	TTAACTGAAACCCTAGCCTCAC GCATCAAGTCAACTCAACTCAA	Marker F
	*IbDCL1b* (Chr 9)	*IbDCL1b_24*	(ATA)6	TTAACTGAAACCCTAGCCTCAC GCATCAAGTCAACTCAACTCAA	Marker G
*IbDCL2*	*IbDCL2b* (Chr 13)	*IbDCL2b_3*	(AGTAAA)2	GCAAGAATCGAATTTAGTGCTC TTCCCGAAATGTCTACTGCTAT	Marker H
	*IbDCL2c* (Chr 6)	*IbDCL2c_2*	(AGTAAA)2	GCAAGAATCGAATTTAGTGCTC TTCCCGAAATGTCTACTGCTAT	Marker I

From the nine heterozygous SSRs on the different chromosomes, we identified 449 alleles in the 50 progenies, among which 51.44% segregated monosomically, 37.27% were co-segregated, and 9.3% fitted the expected disomic inheritance model; trisomic and nullisomic segregation was low (1.55 and 0.44%, respectively) (Table 4). There was deviation (P ≤ 0.01) from the disomic inheritance model for segregation of all markers (Table 4).

**Table 4:** Test for progeny segregation of putative virus defense gene SSRs in a population of 50 seed progeny crosses between ‘New Kawogo’ and ‘Resisto’.

Marker	Model 1:2:1	Progeny deviation from model/alternate models				Test for deviation from the disomic model (Chi-square)

	%Disomic(2n+1)	%Co-segregation (2n)	%Nullisomic (2n-2)	%Monosomic (2n-1)	%Tetrasomic (2n+2)	
A	4 (2)	(48) 96	-	-	-	16^*^
B	16 (8)	(13) 26	-	(27) 54	(2) 4	41^*^
C	28 (14)	(30) 60	-	(3) 6	(3) 6	15^*^
D	24 (12)	(30) 60	-	(6) 12	(2) 4	18.667^*^
E	2 (1)	(4) 8	(2) 4	(43) 86	-	67.667^*^
F	4 (2)	(14) 28	-	(34) 68	-	50^*^
G	2 (1)	(14) 28	-	(35) 70	-	51.33^*^
H	-	(7) 14	-	(43) 86	-	59.669^*^
I	4 (2)	(8) 16	-	(41) 82	-	57.333^*^
Total	9.3 (42)	(168) 37.25	(2) 0.44	(232) 51.44	(7) 1.55	-

Data in parentheses are number of progenies. * Segregation of each marker deviated from the fitted model (Chi-square values for 50 progenies) at P ≤ 0.01.

Inheritance of the defense gene SSRs varied within the progenies, as indicated by the different models of segregation for the markers (Table 4). Inheritance models of markers were found as follows: B, D, and C were disomic, co-segregation, monosomic, and trisomic; E was disomic, co-segregation, nullisomic, and monosomic; F, G, and I were disomic, co-segregating, and monosomic; A was disomic and co-segregating; and H was cosegregating and monosomic. Co-segregation inheritance dominated for markers A, D, and C, while monosomic inheritance dominated for markers B, I, and E. Marker A had the highest proportion of co-segregating progenies (96%), while marker E had the lowest proportion of nullisomic progenies (2%) (Table 4).

## DISCUSSION

Using *in silico* predictions from the sweet potato genome (Yang et al., 2017; Wu et al., 2018), we identified sweet potato putative defense genes, their variants and their microsatellites (SSR markers) and evaluated their segregation patterns. This is the first study of SSRs from specific chromosome locations, gene coding or involved in virus RNA silencing, and their segregation as potential virus defense gene markers in sweet potato progenies. Eight putative defense genes were derived using high and low stringency cut-off values; low stringency prediction has previously been used to derive resistance genes in sugarcane (Wanderley-Nogueira et al., 2007) and to identify defense gene variants (Table 1) in *I. trifida* and *I. triloba* (Wu et al., 2018), *I. nil* (Morgulis et al., 2008), and potato (Hunter et al., 2016). The defense genes of sweet potato were phylogenetically related to defense genes in other plants (Appendix Figures 1 to 8). Specifically, there was close relationship within *Ipomoea* spp. This is confirmed by a related report that was made by Feng et al., (2018) about the evolutionary relationship between *I. batatas* (sweet potato) and wild relatives *I. trifida* and *I. triloba*.

In the present study, the detection of microsatellites (SSR markers) within the DNA coding regions of the defense genes indicates an improvement in understanding of defense genes and virus resistance compared with previous knowledge (Mwanga et al., 2002; Yada et al., 2017). It is important to note that the sweet potato SSRs currently known (Parado, 2010; Wang et al., 2011) are randomly located within the genome and tend to be difficult to develop or study without the use of sophisticated equipment (Schafleitner et al., 2010; Wang et al. 2011); however, the method used in the present is inexpensive and targeted to specific genes and chromosomes. It was found that pentanucleotide repeats were the most abundant (52.04%), followed by hexanucleotide repeats (20.49%) (Table 2). In contrast, hexanucleotide repeats are most frequent (46.38%) in arum lily (*Zantedeschia aethiopica*), followed by monorepeats (31.86%) (Radhika et al., 2011), trinucleotide repeat motifs dominate in citrus and jatropha (Wen et al., 2010), and di-nucleotide repeats dominate in potato (Tang et al., 2009). This study is the first to report the presence of all major forms of repeat motif (mononucleotide, dinucleotide, trinucleotide, tetranucleotide, pentanucleotide, and hexanucleotide) within *IbRDR1*, demonstrating a considerable increase in the number of available genetic markers for sweet potato.

Segregation of putative sweet potato defense genes was analyzed in progenies, and found it tended to be disomic, co-segregating, nullisomic, monosomic, and tetrasomic (Table 4), confirmed by deviations from the expected Mendelian segregation ratio of 1:2:1 (P ≤ 0.01). This deviation may be complex, because no clear pattern of segregation of defense genes was found in sweetpotato. In a study of progeny from different parental crosses, Mwanga et al. (2002) reported that resistance genes segregate in both Mendelian and non-Mendelian patterns, and we suggest this may have occurred in the present study (Table 4). The present results is in contrast with those reported by Rukarwa et al., (2013) for segregation of the *cry7Aa1* gene for weevil resistance in sweetpotato, which segregated in a Mendelian pattern, as would usually be expected for a transgene. When each marker was considered, some progenies inherited genes disomically and fitted well to the Mendelian segregation model (Table 4), indicating almost perfect crossing, whereas other progenies had chromosome doubling (1.55% tetrasomic inheritance) or reduction (0.44% nullisomic inheritance). Interestingly, varied forms of segregation were found within a marker in different progenies (Table 4), indicating that marker segregation in sweet potato may be highly variable among progenies. It is also possible that the allelic composition of a particular defense gene varies among progenies, where it could be an underlying factor in the variability of reversion potential in different sweet potato genotypes (Wasswa et al., 2011; Gibson et al., 2014) and in the variability of disease and pest resistance in potato (Yermishin et al., 2016). Variable patterns of segregation in progenies may also be attributed to segregation distortion of different genes and chromosomes during crossing, as has been reported for barley (Liu et al., 2011) and coffee (Ky et al., 2000), possibly because the large sweet potato chromosome number (2n=6x=90) may be subject to segregation distortion and a high level of cross incompatibility (Knox and Ellis, 2002; Yamagishi et al., 2010).

The breeding of provitamin A-rich orange-fleshed sweet potato with virus resistance is a priority in East Africa (Low et al., 2017). There are, therefore, immediate opportunities for use of this resistance marker gene technique in crop breeding, as demonstrated here, that includes crossing parents with important characteristics (virus resistant, white flesh „New Kawogo‟ and virus susceptible, orange flesh „Resisto‟). The approaches used here may be easily applied to SSR markers for the provitamin A synthetic pathway (Wu et al., 2018) for further development of sweet potato cultivars.

## CONCLUSION

Identification of putative virus resistance genes in the sweet potato genome and development of SSR markers using bioinformatics tools is potentially more efficient than using traditional methods. The SSRs detected in this study may be used in molecular breeding and development of resistance gene analogs, and gene clustering studies of this culturally and economically important crop. This detection of important defense genes in polyploid sweet potato suggests this may be equally possible for other complex genomes, like those of potato and peanut.

## Appendix

Phylograms showing evolutionary relationships of defense genes of sweet potato to other plant species.

## References

[cit0001] Abad JA, Moyer JW (1992). Detection and distribution of sweet potato feathery mottle virus in sweet potato by in vitro transcribed RNA probes (riboprobes), membrane immunobinding assay and direct blotting. Phytopathology 82:300-305.

[cit0002] Addinsoft (2017). Data analysis and statistical solutions for Microsoft Excel. Addinsoft, Paris, France.

[cit0003] Adikini S, Mukasa SB, Mwanga ROM, Gibson RW (2016). Effects of sweet potato feathery mottle virus and sweet potato chlorotic stunt virus on yield of sweet potato in Uganda. Journal of Phytopathology 164:242-254.

[cit0004] Altschul SF, Madden TL, Schaffer AA, Zhang J, Zhang Z, Miller W, Lipman DJ (1997). Gapped BLAST and PSI-BLAST: a new generation of protein database search programs. Nucleic Acids Research 25:3389-3402. 9254694 10.1093/nar/25.17.3389 PMC146917

[cit0005] Baulcombe D (2004). RNA silencing in plants. Nature 431:356-363. 15372043 10.1038/nature02874

[cit0006] Burge CB (1998). Modeling dependencies in pre-Mrna splicing signals. In Salzberg S, Searls D, Kasif S eds. Computational methods in Molecular Biology. Elsevier Sceience, Amsterdam pp. 127-163.

[cit0007] Chellappan P, Vanitharani R, Fauquet CM (2004). Short interfering RNA accumulation correlates with host recovery in DNA virus-infected hosts, and gene silencing targets specific viral sequences. Journal of Virology 78:7465-7477. 15220420 10.1128/JVI.78.14.7465-7477.2004 PMC434130

[cit0008] Edgar RC (2004). MUSCLE: Multiple sequence alignment with high accuracy and high throughput. Nucleic Acid Research 32:1792-1797. 10.1093/nar/gkh340 PMC390337 15034147

[cit0009] Feng JY, Li M, Zhao S, Zhang C, Yang ST, Qiao S, Tan WF, Qu HJ, Wang DY, Pu ZG (2018). Analysis of evolution and genetic diversity of sweet potato and its related different polyploidy wild species I. trifida using RADseq. BioMed Central Plant Biology 18:181. 30185158 10.1186/s12870-018-1399-x PMC6126004

[cit0010] Fuentes S (2010). Sweet potato virus indexing procedure - OP23. International Potato Center (CIP).

[cit0011] Jukes TH, Cantor CR (1969). Evolution of protein molecules. In Munro HN, editor, Mammalian Protein Metabolism, Academic Press, New York pp. 21-132.

[cit0012] Gasura E, Mukasa SB (2010). Prevalence and implications of sweet potato recovery from sweet potato virus disease in Uganda. African Crop Science Journal 18:195-205.

[cit0013] Gibb KS, Podovan AC (1993). Detection of sweet potato feathery mottle virus in sweetpotato grown in northern Australia using an efficient and simple assay. International Journal of Pest Management 39:223-228.

[cit0014] Gibson RW, Kreuze JF (2014). Degeneration in sweet potato due to viruses, virus-cleaned planting material and reversion: a review. Plant Pathology 64:1-15.

[cit0015] Gibson RW, Mpembe J, Alicia T, Carey EE, Mwanga ROM, Seal SE, Vetten HJ (1998). Symptoms, etiology and serological analysis of sweet potato virus diseases in Uganda. Plant Pathology 47:95-102.

[cit0016] Gibson RW, Wasswa P, Tufan HA (2014). The ability of cultivars of sweet potato in East Africa to „revert‟ from sweet potato feathery mottle virus infection. Virus Research 186:130-134. 24361352 10.1016/j.virusres.2013.12.006

[cit0017] Goodstein DM, Shu S, Howson R, Neupane R, Hayes RD, Fazo J, Mitros T, Dirks W, Hellsten U, Putnam N, Rokhsar DS (2012). Phytozome: A comparative platform for green plant genomics. Nucleic Acid Research 40:1178-1186. 10.1093/nar/gkr944 PMC3245001 22110026

[cit0018] Green SK, Kuo YJ, Lee DR (1988). Uneven distribution of two potyviruses (sweet potato feathery mottle virus and sweet potato latent virus) in sweet potato plants and its implication on indexing of meristem-derived plants. Tropical Pest Management 34:298-302.

[cit0019] Griffing B (1956). Concept of general and specific combining ability in relation to diallel crossing systems. Australian Journal of Biological Sciences 9:463-493.

[cit0020] Guo Y, Wu Y, Anderson JA, Moss JQ, Zhu L (2015). Disomic inheritance and segregation distortion of SSR markers in two populations of *Cynodon dactylon* (L.) Pers. var. dactylon. PLoS ONE 10:e0136332. 26295707 10.1371/journal.pone.0136332 PMC4546580

[cit0021] Hunter LJR, Brockington SF, Murphy AM, Pate AE, Gruden K, MacFarlane SA, Palukaitis P, Carr JP (2016). RNA-dependent RNA polymerase 1 in potato (*Solanum tuberosum*) and its relationship to other plant RNA dependent RNA polymerases. Scientific Reports 6:23082. 26979928 10.1038/srep23082 PMC4793286

[cit0022] Knox MR, Ellis THN (2002). Excess heterozygosity contributes to genetic map expansion in pea recombinant inbred populations. Genetics 162:861-873. 12399396 10.1093/genetics/162.2.861 PMC1462271

[cit0023] Ky CL, Barre P, Lorieux M, Trouslot P, Akaffou S, Louarn J, Charrier A, Hamon S, Noirot M (2000). Interspecific genetic linkage map, segregation distortion and genetic conversion in coffee (*Coffea sp*.). Theoretical and Applied Genetics 101:669-676.

[cit0024] Leibman D, Kravchik M, Wolf D, Haviv S, Weissberg M, Ophir R, Paris HS, Palukaitis P, Ding SW, Gaba V, Gal-on A (2017). Differential expression of cucumber RNA-dependent RNA polymerase 1 genes during antiviral defence and resistance. Molecular Plant Pathology 19:300-312. 27879040 10.1111/mpp.12518 PMC6637986

[cit0025] Liu X, You J, Guo L, Liu X, He Y, Yuan J, Liu G (2011). Genetic analysis of segregation distortion of SSR markers in F2 population of barley. Journal of Agricultural Science 3:172-177.

[cit0026] Lomsadze A, Gemaye K, Tang S, Borodovsky M (2018). Modeling leaderless transcription and atypical genes results in more accurate gene prediction in prokaryotes. Genome Research 28:1079-1089. 29773659 10.1101/gr.230615.117 PMC6028130

[cit0027] Low JW, Mwanga ROM, Andrade M, Carey E, Ball AM (2017). Tackling vitamin A deficiency with biofortified sweet potato in sub-Saharan Africa. Global Food Security 14:23-30. 28989861 10.1016/j.gfs.2017.01.004 PMC5614018

[cit0028] Martins WS, Lucas DCS, de Souza Neves KF, Bertioli DJ (2009). WebSat - A web software for microsatellite marker development. Bioinformation 3:282-283. 19255650 10.6026/97320630003282 PMC2646864

[cit0029] Maruthi MN, Colvin J, Seal S, Gibson G, Cooper J (2002). Coadaptation between cassava mosaic geminiviruses and their local vector populations. Virus Research 86:71-85. 12076831 10.1016/s0168-1702(02)00051-5

[cit0030] Mason AS (2015). SSR Genotyping. In: Batley J, editor. Plant Genotyping. Springer; New York, NY: pp. 77–89.

[cit0031] Morgulis AR, Coulouris G, Raytselis Y, Madden TL, Agarwala R, Schafffer AA (2008). Database indexing for production MegaBLAST searches. Bioinformatics 24:1757-1764. 18567917 10.1093/bioinformatics/btn322 PMC2696921

[cit0032] Mukasa SB, Rubaihayo PR, Valkonen JPT (2003). Incidence of viruses and virus like diseases of sweet potato in Uganda. Plant Disease 87:329-335. 30831824 10.1094/PDIS.2003.87.4.329

[cit0033] Mwanga ROM, Kriegner A, Cervantes-Flores JC, Zhang DP, Moyer JW, Yencho GC (2002). Resistance to sweet potato chlorotic stunt virus and sweet potato feathery mottle virus is mediated by two separate recessive genes in sweet potato. Journal of the American Society for Horticultural Science 127:798-806.

[cit0034] Mwanga ROM, Kyalo G, Ssemakula GN, Niringiye C, Yada B, Otema MA (2016). NASPOT 12 O” and “NASPOT 13 O” sweetpotato. HortScience 51:291-295.

[cit0035] Mwanga ROM, Odongo B, Niringiye C, Alajo A (2007). Release of two orange-fleshed sweet potato cultivars, „SPK004‟ („Kakamega‟) and „Ejumula‟, in Uganda. HortScience 42:1728-1730.

[cit0036] Mwanga ROM, Ssemakula G (2011). Orange-fleshed sweetpotatoes for food, health and wealth in Uganda. International Journal of Agricultural Sustainability 9:42-49.

[cit0037] Mueller LA, Solow TH, Taylor N, Skwareck B, Buels R, Binns J, Lin C, Wright MH, Ahrens R, Wang Y, Herbst EV, Keyder ER, Menda N, Zamir D, Tanksley SD (2005). The SOL Genomics Network: a comparative resource for Solanaceae biology and beyond. Plant Physiology 138:1310-1317. 16010005 10.1104/pp.105.060707 PMC1176404

[cit0038] Okonechnikoy K, Golosova O, Fursov M (2012). The UGENE team. Unipro UGENE: a unified bioinformatics toolkit. Bioinformatics 28:1166-1167. 22368248 10.1093/bioinformatics/bts091

[cit0039] Parado KH (2010). SSR for sweetpotato. www.Research.cip.cgiar.orgResearch.cip.cgiar.org. Accessed 2/11/2018.

[cit0040] Peragine A, Yoshikawa M, Wu G, Albrecht HL, Poethig RS (2004). SGS3 and SGS2/ SDE1/RDR6 are required for juvenile development and the production of trans-acting siRNAs in Arabidopsis. Genes and Development 18:2368-2379. 15466488 10.1101/gad.1231804 PMC522987

[cit0041] Puelma T, Araus V, Canales J, Vidal E, Cabello J, Soto A, Gutierrez R (2017). GENIUS: Web server to predict local gene networks and key genes for biological functions. Bioinformatics 33:760-761. 27993775 10.1093/bioinformatics/btw702 PMC5408817

[cit0042] Rachkara P, Phillips DP, Kalule SW, Gibson RW (2017). Innovative and beneficial informal sweet potato seed private enterprise in northern Uganda. Food Security 9:595-610. 32968464 10.1007/s12571-017-0680-4 PMC7473088

[cit0043] Radhika V, Aswath C, Lakshman Reddy DC, Bhardwaj SA (2011). *In silico* microsatellite development in arum lily (*Zantedeschia aethiopica*). Journal of Horticultural Science 6:37-40.

[cit0044] Rukarwa RJ, Mukasa SB, Sefasi A, Ssemakula G, Mwanga ROM, Ghislain M (2013). Segregation analysis of cry7Aa1 gene in F1 progenies of transgenic and non-transgenic sweet potato crosses. Journal of Plant Breeding and Crop Science 5:209-213.

[cit0045] Schafleitner R, Tincopa LR, Palomino O, Rossel G, Robles RF, Alagon R, Rivera C, Quispe C, Rojas L, Pacheco JA, Solis J, Cerna D, Kim JY, Hou J, Simon R (2010). A sweet potato gene index established by denovo assembly of pyrosequencing and Sanger sequences and mining for gene-based microsatellite markers. BMC Genome 11:604. 10.1186/1471-2164-11-604 PMC3017860 20977749

[cit0046] Stanke M, Steinkamp R, Waack S, Morgenstern B (2004). AUGUSTUS: A web server for gene finding in eukaryotes. Nucleic Acids Research 32 (Web Server issue) W309-12. 15215400 10.1093/nar/gkh379 PMC441517

[cit0047] Stift M, Berenos C, Kuperus P, van Tienderen PH (2008). Segregation models for disomic, tetrasomic and intermediate inheritance in tetraploids: A general procedure applied to *Rorippa* (Yellow Cress) microsatellite data. Genetics 179:2113-2123. 18689891 10.1534/genetics.107.085027 PMC2516083

[cit0048] Tang S, Okashah RA, Cordonnier-Pratt M, Prat LH, Johnson VE, Taylor CA, Arnold ML, Knapp SJ (2009). EST and EST-SSR marker resources for Iris. BMC Plant Biology 9:72. 19515254 10.1186/1471-2229-9-72 PMC2703627

[cit0049] Vieira MLC, Santini L, Diniz AL, Munhoz CF (2016). Microsatellite markers: what they mean and why they are so useful. Genetics and Molecular Biology 39:312-28. 27561112 10.1590/1678-4685-GMB-2016-0027 PMC5004837

[cit0050] Wanderley-Nogueira AC, Soares-Cavalcanti NM, Morais DAL, Belarmino LC, Barbosa-Silva A, Benko-Iseppon AM (2007). Abundance and diversity of resistance genes in the sugarcane transcriptome revealed by in silico analysis. Genetics and Molecular Research 6:866-889. 18058709

[cit0051] Wang Z, Li J, Luo Z, Huang L, Chen X, Fang B, Li Y, Chen J, Zhang X (2011). Characterization and development of EST-derived SSR markers in cultivated sweet potato (*Ipomoea batatas*). BMC Plant Biology 11:139. 22011271 10.1186/1471-2229-11-139 PMC3206431

[cit0052] Wasswa P, Otto B, Maruthi MN, Mukasa SB, Monger W, Gibson RW (2011). First identification of a sweet potato begomovirus (sweepovirus) in Uganda: characterization, detection and distribution. Plant Pathology 60:1030-1039.

[cit0053] Wheeler DL, Church DM, Federhen S, Lash LE, Madden TL, Pontius JU, Schuler GD, Schriml LM, Sequeira E, Tatusova TA, Wagner L (2003). Database resources of the National Center for Biotechnology. Nucleic Acid Research 31:28-33. 10.1093/nar/gkg033 PMC165480 12519941

[cit0054] Wen M, Wang H, Xia Z, Zou M, Lu C, Wang W (2010). Development of EST-SSR and genomic-SSR markers to assess genetic diversity in *Jatropha curcas* L. BMC Research Notes 3:42. 20181259 10.1186/1756-0500-3-42 PMC2844395

[cit0055] Wu S, Lau KH, Cao Q, Hamilton JP, Sun H, Zhou C, Eserman L, Gemenet D, Olukolu B, Wang H, Crisovan E, Godden GT, Jiao C Wang X, Kitavi M, Manrique-Carpintero N, Vaillancourt B, WiegertRininger K, Yang X, Bao K, Zheng Y, Schaff J, Kreuze J, Gruneberg W, Khan A, Ghislain M, Ma D, Jiang J, Mwanga ROM, Leebens-Mack J, Coin LJM, Yencho GC, Buell CR, Fei Z (2018). Genome sequences of two diploid wild relatives of cultivated sweet potato reveal targets for genetic improvement. Nature Communications 9:4580. 10.1038/s41467-018-06983-8 PMC6214957 30389915

[cit0056] Yada B, Alajo A, Ssemakula GN, Mwanga ROM, Brown-Guedira G, Yencho GC (2017). Selection of Simple Sequence Repeat Markers Associated with Inheritance of Sweet potato Virus Disease Resistance in Sweetpotato. Crop Science 57:1421-1430.

[cit0057] Yamagishi M, Takeuchi Y, Tanaka I, Kono I, Murai K, Yano M (2010). Segregation distortion in F2 and doubled haploid populations of temperate japonica rice. Journal of Genetics 89:237-241. 20861577 10.1007/s12041-010-0032-z

[cit0058] Yang J, Moeinzadeh MH, Kuhl H, Helmuth J, Xiao P, Haas S, Liu G, Zheng J, Sun Z, Fan W, Deng G (2017). Haplotype-resolved sweet potato genome traces back its hexaploidization history. Nature Plants 3:696-703. 28827752 10.1038/s41477-017-0002-z

[cit0059] Yeh RF, Lim LP, Burge CB (2001). Computational inference of homologous gene structures in the human genome. Genome Research 11:803-816. 11337476 10.1101/gr.175701 PMC311055

[cit0060] Yermishin AP, Svitoch OV, Voronkova EV, Gukasian ON, Luksha VI (2016). Determination of the composition and the allelic state of disease and pest resistance genes in potato parental lines using DNA markers. Russian Journal of Genetics 52:498-506. 29368481

[cit0061] Zou JJ, Singh RJ, Lee J, Xu SJ, Hymowitz T (2006). SSR markers exhibit trisomic segregation distortion in soybean. Crop Breeding and Genetics 46:1456-1461.

